# Elements of Anatomy

**Published:** 1846-04

**Authors:** 


Art. VII.—*Elements of Anatomy.* By Jones Quain, m.d. Fifth Edition. Edited by Richard Quain and William Sharpey, m.d., Professors of Anatomy and Physiology, University College, London. Illustrated by numerous engravings on wood. Parts I, II.—*London,* 1843, 1846. 8vo, pp. 1063.

It would seem as if the very fact of the division of a work into parts, and the separate publication of these, exerted an injurious influence on the minds of the parties concerned, inducing procrastination and forgetfulness of promises, and consequently disappointing all the hopes which credulous buyers had founded on the promises liberally put forth in the first announcement. It has certainly become the *exception,* rather than the *rule,* for a work published in parts to be completed in anything like the time originally intended; and the work before us cannot, we regret to say, claim exemption from the censure we have heretofore administered in similar cases. In fact, we are constrained to remark, that we think there is less excuse when a new edition of a work previously complete is concerned than where the entire work is new. Surely the editors might form some tolerable estimate, in the first instance, of the amount of alteration that would be required, and might withhold the publication of any part until the remainder were in an advanced condition. The first part of this new edition of Dr. Quain's Anatomy was issued in the autumn of 1843, with the promise that the remainder should be published "as soon as possible." The second part has only made its appearance within these few weeks, consequently, nearly two years and a half after the first, and it does not complete the work. By way of apology, we are told that "the changes made are much more extensive than had been originally contemplated. In fact the principal part of this volume has been written anew." Our question is,—why were not these changes *originally* contemplated,—or, in other words, why did not the editors decide upon the amount of change they would introduce *before* they proceeded with the first part? Anatomy is not a science of such rapid progress as to require the complete remodelling of a previously good treatise every three or four years; and we cannot but think, that such highly-qualified editors might have more clearly divined their path at the outset of their course, and that, even if they found it requisite to deviate from it here and there, the delay need not have been quite so long. The evil is much greater in regard to a work especially destined for the student, than with respect to one which is chiefly intended for the established practitioner. The youth who purchased the first part, as the guide of his anatomical studies,—presuming, as he might fairly do in such a case, that the remainder would appear within the session, or at furthest at the commencement of the next session,—after spending three or four years in attendance on lectures, finds himself still the possessor of an incomplete work, and the last part makes its appearance just when it is becoming comparatively useless to him.

It pains us greatly to be continually obliged to recur to this subject, by the misdoings of medical authors; more particularly when we have to direct our censure upon those who in every other respect deserve the highest praise at our hands. We have no hesitation in saying that the work before us, completed (as we feel confident it will be) in the spirit in which it has been thus far carried on, will be alike worthy of the fame of its distinguished editors, and valuable to the anatomical student. The former editions of Dr. Quain's 'Elements' had attained a high reputation, as having successfully avoided the useless minuteness of some treatises, and the superficial curtness of others; laying deserved stress upon those points which enter into the consideration of practical questions, guiding the physician in his diagnosis and treatment, and directing the judgment and hand of the surgeon, and, at the same time, giving all needful information upon other matters of less obvious utility. The chief advances made, since the publication of the fourth edition, have been in the department of general anatomy. The whole of this has been rewritten by Dr. Sharpey, and forms an introduction, separately paged, illustrated by a large number of admirably executed wood engravings. It is now a most admirable and concise exposition of that subject; and we should much like to see it issued separately, for the sake of those of our brethren more advanced in life, who desire to make themselves acquainted with what has been recently effected by the aid of the microscope and of chemical analysis, in regard to the elementary structure of the tissues, and who do not require to possess themselves of a general treatise on anatomy. The purely anatomical portion of the work has been chiefly revised by Mr. Quain; but we think we discern, in the alterations which have been made in the account of the nervous centres, the indications of Dr. Sharpey's physiological mode of treating this difficult subject; at any rate, this portion of the work is remarkably well done.

We shall give a more detailed account of the work when it shall be completed, by which time we hope that Messrs. Todd and Bowman's Physiological Anatomy may be in the same condition, so that we may review them together, and point out their respective excellencies.

